# Deep learning-based gait phase detection using shank-mounted IMU data: Classification approach

**DOI:** 10.1371/journal.pone.0344002

**Published:** 2026-04-15

**Authors:** Wonseok Choi, Mun-Taek Choi

**Affiliations:** Department of Intelligent Robotics, Sungkyunkwan University, Suwon, South Korea; Beijing Institute of Technology, CHINA

## Abstract

Gait is a key indicator for assessing an individual’s mobility and overall health, and accurate detection of gait cycle phases is essential for precise gait analysis. Most existing gait phase detection approaches rely on multiple wearable sensors, increasing system complexity and limiting real-world applicability. Moreover, rule-based methods, traditional machine learning models, and convolutional neural network (CNN)-based approaches often fail to capture complex temporal dependencies, resulting in limited robustness and real-time performance. And approaching gait phase detection as a classification method enables automatic phase recognition directly from sequential signals, simplifying the processing pipeline and supporting stable performance. In this study, we utilized an end-to-end supervised classification learning approach using a state-of-the-art Transformer model. We utilized the publicly available NONAN GaitPrint dataset, time-series gait recordings from 35 healthy young adults who walked along a 200-meter indoor level-ground track, to train deep learning models for gait phase detection. Using the three-axis acceleration and three-axis angular velocity signals from the shank, we trained and evaluated a variety of classification models, including a one-dimensional CNN, a hybrid long short-term memory and gated recurrent unit (Hybrid LSTM+GRU) model, and a Transformer. The results showed that the Transformer model achieved the F1-score of approximately 92.99%, while the CNN and Hybrid LSTM+GRU models demonstrated comparable performance, indicating no substantial differences among the models. These findings demonstrate that clinically and practically feasible gait phase detection can be achieved using shank-mounted IMUs with an end-to-end learning approach.

## Introduction

Human gait is a primary mode of locomotion that involves a complex interplay of biomechanical and neuromuscular components [1]. To quantitatively analyze gait, it is essential to divide the gait cycle into sequential intervals and define the functional characteristics of each segment—commonly referred to as gait phases [2]. A typical gait cycle consists of the following phases: Loading Response (LR), where the body absorbs impact following heel contact; Mid Stance (MS) and Terminal Stance (TS), which correspond to periods of single-limb support; Pre-Swing (PSw), the transition phase prior to toe-off; and Swing (Sw), during which the limb is airborne and progresses in the forward direction. These gait phases serve as fundamental units that describe the temporal progression of the gait cycle, and are widely used to quantify gait continuity, stability, and functional changes [3,4]. Accurate classification of gait phases is critical in various applications, including clinical diagnostics, rehabilitation therapy, and real-time monitoring systems using wearable sensors [5–7].

Accurate classification of gait phases requires detailed analysis of the gait cycle, and a variety of measurement and analytical techniques have been proposed to achieve this. Common methods for collecting gait data include camera-based 3D motion capture systems, instrumented treadmills, and wearable sensor systems [8,9]. However, these approaches often involve high equipment costs, complex setup procedures, and limitations to controlled laboratory environments [10]. In contrast, inertial measurement units (IMUs) are increasingly utilized for gait analysis, given their low cost, portability, and practicality in everyday settings [11–13]. Nevertheless, many previous studies have relied on attaching multiple IMUs to various body segments in order to accurately segment gait phases. This approach increases the burden on the user, raises the overall equipment cost, and reduces feasibility for field applications [14–16]. To overcome these limitations, recent research has focused on classifying gait phases with a minimal number of sensors, particularly with single-IMU configurations, which aim to reduce user discomfort while maintaining high classification performance [17–19].

The shank segment is known to exhibit the most distinctive movement patterns during walking, and changes in acceleration and angular velocity are clearly observable at this location [20–22]. Signals measured by IMU sensors—specifically acceleration and angular velocity—sensitively reflect both linear and rotational movements during gait, with notable fluctuations, directional shifts, and periodic differences occurring especially at phase transition points [23,24]. For this reason, shank-mounted IMUs are often sufficient to capture the essential discriminative features required for gait phase classification [25]. This makes it particularly suitable for deep learning-based time-series classification models, which can effectively learn the structural characteristics of each gait phase from raw sensor signals.

Previous studies on gait phase analysis have primarily employed regression-based approaches [26] or classification-based approaches [27], utilizing various machine learning methods such as k-nearest neighbors, support vector machines, and hidden Markov models [28–32]. While these traditional methods offer advantages such as relatively low computational cost and interpretable structures, they often struggle to capture the temporal dynamics of gait phases when the input data are nonlinear and feature boundaries are ambiguous due to the complexity of human movement and inter-individual variability.

Recent studies have increasingly applied deep learning techniques to IMU signals for gait phase classification, demonstrating that deep models can effectively capture temporal patterns and salient features across various walking conditions [33,34]. Building on this trend, deep learning models can autonomously extract high-level representations—such as temporal patterns, periodicity, and phase transition characteristics—from raw time-series inputs, achieving high classification accuracy without explicit feature engineering [35]. This capability enables effective learning of the temporal structure inherent in gait signals and has been shown to yield robust performance even under real-world measurement environments.

In this study, we use gait data from healthy adults collected via IMU sensors attached to both shanks, employing three-axis acceleration and angular velocity signals to evaluate deep learning–based gait phase classification. Gait phases are labeled by extracting gait events from foot-contact data and assigning the corresponding phase to each time frame. We adopt an end-to-end learning framework without hand-crafted features, allowing the models to directly learn temporal dynamics and phase-transition characteristics from raw time-series inputs. To examine how well gait phases can be identified under a minimal-sensor configuration, we evaluate three representative deep learning architectures—a convolutional neural network (CNN), a hybrid long short-term memory and gated recurrent unit (Hybrid LSTM+GRU), and a Transformer encoder. The results show that all three models achieve consistent and reliable performance using only shank-mounted IMU data, indicating that shank-mounted IMUs provides sufficient temporal information for multi-class gait phase classification in level-ground walking. By comparing these architectures under identical experimental conditions, this study clarifies the strengths of lightweight models for simple, highly periodic gait patterns and highlights the advantages of more advanced architectures for scenarios involving greater temporal complexity. Overall, this work demonstrates that minimal-sensor deep learning approaches can substantially reduce system complexity while maintaining clinically and practically meaningful gait phase recognition performance.

## Materials and methods

### Dataset

**Fig 1:**
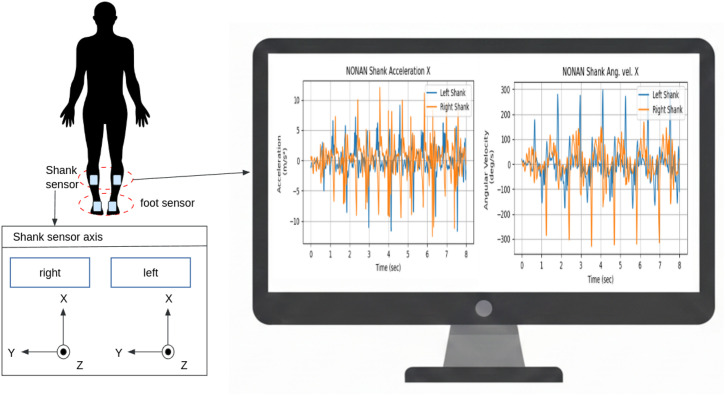
Depiction of IMU sensor positioning on the shank and foot, along with sensor axes and representative signals. The foot-mounted sensor was used to obtain contact data.

In this study, we utilized the publicly available NONAN GaitPrint dataset [36] to train and evaluate deep learning models for gait phase detection. The dataset comprises time-series gait recordings from 35 healthy young adults who walked along a 200-meter indoor track. Each participant completed 18 trials (4 minutes per trial) over two separate days, with data that had been collected from multiple joints using Noraxon Ultium Motion IMU sensors, sampled at 200 Hz. Table 1 provides the key features and structure of the NONAN GaitPrint dataset. For this study, the IMU data were resampled to 100 Hz to improve computational efficiency and reduce overfitting. We used acceleration and angular velocity along three axes from IMU sensors mounted on both shanks. Fig 1 illustrates the placement of the sensors and visualizes one representative axis from both acceleration and angular velocity signals.

**Table 1:** Overview of NONAN GaitPrint dataset characteristics.

Feature	Mean ± Std Dev	Range
Age (years)	24.6 ± 2.7	19–35
Height (m)	1.73 ± 0.78	–
Weight (kg)	72.44 ± 15.04	42.3–91
Male participants	18 (51.43%)	–
Female participants	17 (48.57%)	–

### Data preprocessing

**Fig 2:**
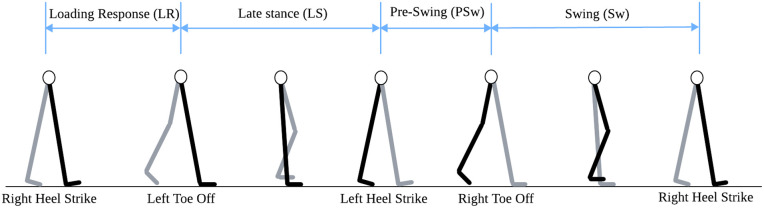
Gait phase transition diagram for a gait cycle. Black indicates the right leg and gray indicates the left leg.

In consideration of the extensive size of the NONAN dataset, and to ensure computational efficiency and practical feasibility of the analysis, we utilized four randomly selected trials per participant out of the total 18 trials. Based on this, prior to performing gait phase labeling, four gait phases were defined as illustrated in Fig 2. Among them, Late Stance (LS) was formed by combining MS and TS into a continuous support phase, since the functional difference between them was regarded as minimal in real-time applications [37]. This integration was intended to simplify the model and enhance its practical applicability.

**Fig 3:**
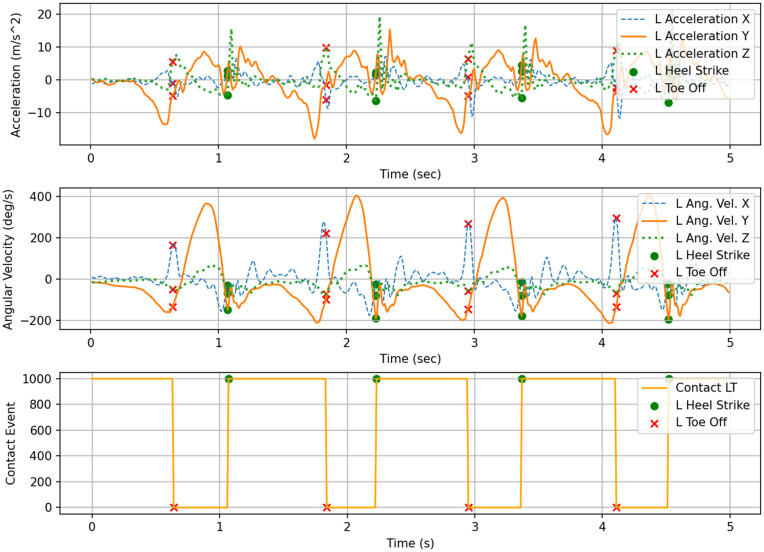
Input signals of left shank: acceleration, angular-velocity, and contact data.

**Fig 4:**
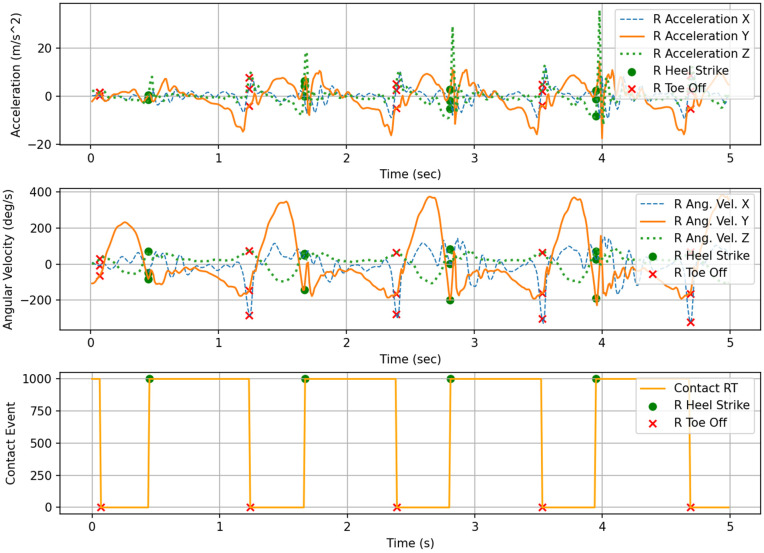
Input signals of right shank: acceleration, angular-velocity, and contact data.

Gait phase labeling was performed by first automatically detecting Heel Strike (HS) and Toe Off (TO) events from the bilateral foot contact signals. Each phase was then defined based on the temporal intervals between these gait events [38]. Figs 3 and 4 illustrate the detected HS and TO event timings from the left and right foot contact signals, providing a clear visualization of the temporal structure in which these events occur within the gait cycle. The gait phases were defined according to the following rules: the interval from right HS to left TO was labeled as the LR, and the interval from left HS to left TO was labeled as the LS. Subsequently, the period from left TO to the next left HS was labeled as the PSw, and the interval from left TO to the next right HS was labeled as the Sw. Based on these rules, gait phase labels were automatically assigned to the entire time-series sequence using both sides’ contact data.

To train and evaluate the deep learning models, we constructed input–output sequences consisting of time-series inputs *x* together with their associated gait phase labels *y*. The input sequence *x* comprises IMU time-series data recorded from both shanks, while *y* represents the corresponding gait phase label at each time step. The dataset contains *n* time steps, with each time point associated with synchronized shank IMU signals and their respective phase labels. To better model the temporal dynamics of gait phases, we designed a sequence-based input structure using a sliding window approach [39]. Table 2 illustrates how the dataset was organized using this method. The window length *w* and the prediction offset are both tunable hyperparameters; in this study, they were fixed to 20 and +1, respectively, to ensure consistent model training and stable sequence representation. Based on the defined window length, each input sequence consists of *w* consecutive IMU measurements $\left(x_{1},x_{2},\ldots ,x_{w}\right)$, and the corresponding output label is taken from the subsequent time step *y*_*w*+1_. This one-step-ahead prediction structure is intended to mitigate potential label latency in real-time time-series applications by predicting the phase at the next time step rather than the final point of the input window. The window then slides forward by one time step to generate the next input–output pair, resulting in a total of n−w training samples. Specifically, at the final time step *n*, the input sequence is $\left(x_{n-w},x_{n-w+1},\ldots ,x_{n-1}\right)$, and the corresponding output label is *y*_*n*_. This structure enables the model to estimate the upcoming gait phase based on preceding temporal information, thereby facilitating the learning of transition dynamics and temporal dependencies within the gait sequence.

**Table 2:** Sliding window representation of training data. Each sample consists of *w* consecutive IMU inputs and the label at the next time step.

Sample No.	Input	Output (label)
1	$x_{1},x_{2},\cdots ,x_{w}$	*y*_*w* + 1_
2	$x_{2},x_{3},\cdots ,x_{w+1}$	*y*_*w* + 2_
3	$x_{3},x_{4},\cdots ,x_{w+2}$	*y*_*w* + 3_
⋮	⋮	⋮
n−w	$x_{n-w},x_{n-w+1},\cdots ,x_{n-1}$	*y*_*n*_

### Deep Learning Models

**Fig 5:**
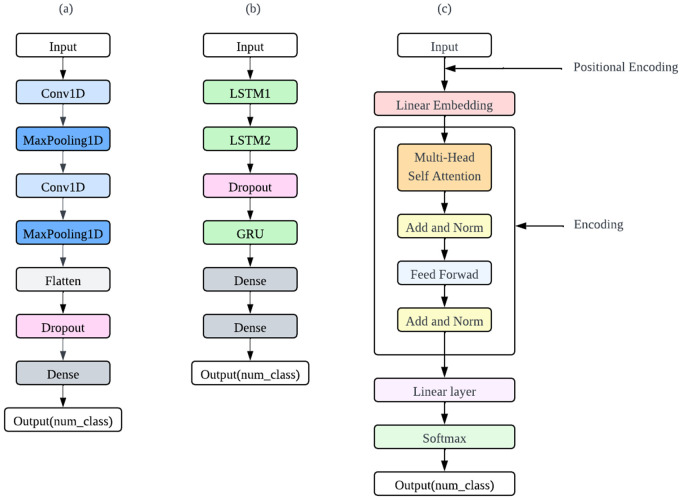
Architectural diagrams for deep learning models employed in gait phase classification. The figure illustrates **(a)** CNN model, **(b)** Hybrid LSTM+GRU model, and **(c)** Transformer encoder model.

To effectively classify gait phases, several deep learning models were utilized, including CNN, Hybrid LSTM+GRU, and Transformer architectures. The structures of these models are illustrated in Fig 5, and their objective is to capture spatial and temporal features from sequential IMU data so as to accurately classify each gait phase within the gait cycle.

The CNN model is based on a one-dimensional CNN, which is well-suited for extracting local spatial patterns from time-series sensor data. The architecture consists of two convolutional layers, followed by pooling layers and fully connected dense layers [40]. The convolution operation can be expressed as:


$${𝐳}_{t}=\sigma \left(\sum_{i=0}^{k-1}{𝐰}_{i}\cdot {𝐱}_{t+i}+b\right)$$

where **x**_*t*+*i*_ represents the *i*-th input vector at time step *t* + *i*, **w**_*i*_ is the filter weight, *b* is the bias term, and σ(·) is a nonlinear activation function.

The hybrid LSTM+GRU model is constructed to leverage both short- and long-term temporal dependencies in the gait signal. It consists of two LSTM layers followed by a GRU layer. The LSTM layers capture long-range temporal features, while the GRU layer refines the sequential representation with improved computational efficiency [41]. The forward computations for the hybrid structure are summarized as follows:


$$\begin{array}{cc} {𝐡}_{t}^{LSTM} & =LSTM({𝐱}_{t},{𝐡}_{t-1}^{LSTM},{𝐜}_{t-1})\\ {𝐡}_{t}^{GRU} & =GRU({𝐡}_{t}^{LSTM},{𝐡}_{t-1}^{GRU}) \end{array}$$

where **x**_*t*_ is the input at time *t*, ${𝐡}_{t}^{LSTM}$ and ${𝐡}_{t}^{GRU}$ denote the hidden states of the LSTM and GRU layers, respectively, and ${𝐜}_{t-1}$ is the previous cell state in the LSTM. Finally, the output of the GRU layer is passed through fully connected layers, and a sigmoid activation function is applied to perform multi-class gait phase classification.

The Transformer encoder is employed in this study to detect gait phases, leveraging its ability to model long-range dependencies and process sequences in parallel [42]. As the task involves predicting a single class label from an input sequence, only the encoder component is used, omitting the decoder. The core mechanism of the encoder is self-attention, which enables each time step to attend to others in the sequence. It is defined as:


$$\text{Attention}(Q,K,V)=\text{softmax}\left(\frac{QK^{⊤}}{\sqrt{d_{k}}}\right)V$$

Here, *Q*, *K*, and *V* represent the query, key, and value matrices, and *d*_*k*_ is the dimension of the key vectors. This operation allows the model to dynamically weight the influence of each time step when forming the output representation.

Each encoder layer consists of three main components. First, multi-head attention allows the model to jointly attend to information from multiple representation subspaces. Second, a position-wise feed-forward network enhances nonlinearity by applying two dense layers to each time step. Third, sinusoidal positional encoding is added to input embeddings to convey order information:


$${\text{PE}}_{\left(pos,2i\right)}=\sin\left(\frac{pos}{10000^{2i/d_{\text{model}}}}\right),\;{\text{PE}}_{\left(pos,2i+1\right)}=\cos\left(\frac{pos}{10000^{2i/d_{\text{model}}}}\right)$$

Through this encoder-only architecture, the Transformer effectively captures global temporal patterns in gait sequences and offers improved computational efficiency over recurrent models, which process data sequentially.

### Training configuration

Data partitioning was performed using a subject-wise split to evaluate how well the models generalize to unseen participants. The training dataset consisted of data from 24 subjects, with three sessions per subject allocated to training and the remaining session split for validation. The test dataset included all four sessions from 11 subjects who were not involved in either training or validation. To normalize the statistical distribution of the time-series data, scikit-learn’s [43] StandardScaler was applied so that each sensor channel had zero mean and unit variance. In addition, to prevent biased learning toward particular classes, the training data were resampled to equalize the sample count of the smallest class [44].

In the model training stage, all models were trained for 80 epochs to ensure a fair comparison. However, certain hyperparameters were applied differently across models to achieve optimal performance. Specifically, the CNN and Hybrid LSTM+GRU models applied a dropout rate of 0.3 and the Adam optimizer with a batch size of 1024, whereas the Transformer model was trained with a dropout rate of 0.4, the AdamW optimizer, and a batch size of 128. Other key hyperparameters were optimized for each model using Optuna prior to evaluation. This experimental design was intended to promote stable convergence under limited training data conditions while enabling a fair and quantitative comparison of performance across models.

## Results

### Hyperparameter

Optuna [45] was employed to perform hyperparameter tuning for each deep learning model, and the results are provided in Table 3. This table presents the explored tuning ranges along with the best hyperparameters, which were selected based on validation accuracy. Using these best parameters, the final test dataset was used to evaluate each deep learning model after training. Each deep learning model, trained with the selected best parameters, is compared using accuracy (ACC) and macro-averaged precision (PR), recall (RC), and F1-score (F1).

**Table 3:** Optuna-based hyperparameter tuning: ranges and best parameters.

Model	Tuning Range	Best Parameter
**CNN**	Number of Filters: [32, 64, 128]	**32**
	Kernel Size: [1,3,5]	**1**
	Learning Rate: [0.00001–0.0005]	**0.0001**
**Hybrid LSTM+GRU**	LSTM Units1: [100, 200]	**100**
	LSTM Units2: [100, 200]	**100**
	GRU Units: [128, 256, 512]	**256**
	Dense Units: [128, 256, 512]	**128**
	Learning Rate: [0.0001–0.01]	**0.0022**
**Transformer**	Embedding Dimension: [128, 256]	**256**
	Multi-Head Attention: [1,2]	**1**
	Encoder Layers: [2,4]	**2**
	Feedforward Dimension: [512, 1024]	**512**
	Learning Rate: [0.00002–0.00005]	**0.0000292**

### Multi-class classification results

**Fig 6:**
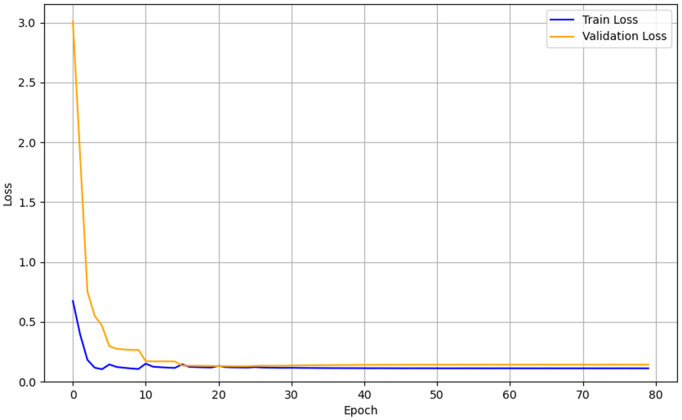
Learning curve of the CNN.

**Fig 7:**
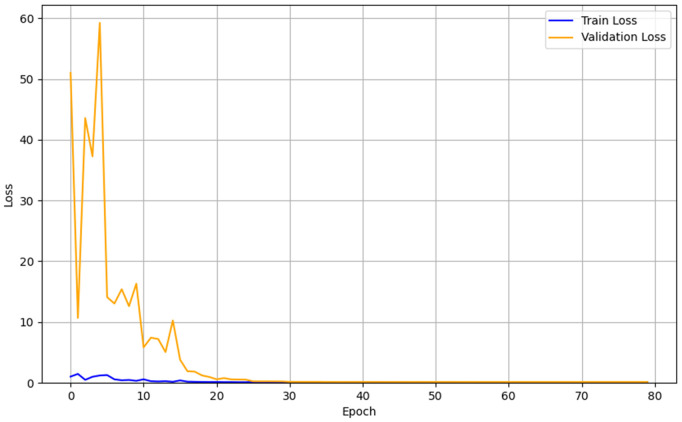
Learning curve of the Hybrid LSTM+GRU.

**Fig 8:**
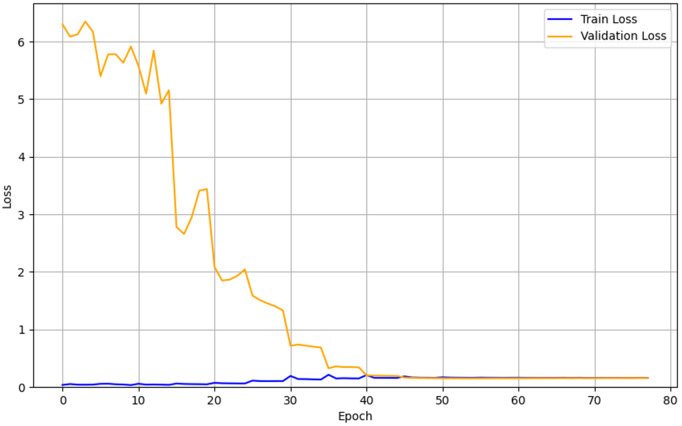
Learning curve of the Transformer.

The analysis of the training and validation loss curves is presented in Figs 6–8. All three models converged stably in the final stages of training, although their early convergence patterns differed. The CNN exhibited a rapid and smooth reduction in loss from the initial epochs, attributed to its strong inductive bias from locality and weight sharing, as well as its relatively small number of parameters, and ultimately converged to a train loss of 0.14 and a validation loss of 0.11. The Hybrid LSTM+GRU showed greater fluctuations in validation loss during the early epochs, reflecting the time required for the initialization and stabilization of recurrent states and gating mechanisms, but eventually stabilized with a train loss of 0.09 and a validation loss of 0.13. The Transformer was more sensitive to parameter scaling and learning rate settings during the early training phase, resulting in relatively larger fluctuations, but gradually stabilized as training progressed, converging to a train loss of 0.158 and a validation loss of 0.155. Since the differences between training and validation losses remained small and the final loss values converged at low levels across all models, no severe overfitting was observed, indicating that the models were trained stably on the given dataset. These patterns illustrate typical learning dynamics, where the initial instability observed in Transformer and Hybrid LSTM+GRU models is eventually resolved into stable convergence, while the CNN, owing to its structural simplicity and strong inductive bias, converged stably from the outset.

The confusion matrices for the test dataset are shown in Tables 4, 5, and 6. Consistent with the stable convergence observed in the learning curves, the models achieved overall balanced classification performance. The confusion matrix was reported using raw counts, and due to the dataset distribution, the number of samples in LS and Sw was about three times larger than in LR and PSw, leading to apparent differences in absolute misclassification counts. However, when interpreted in terms of relative proportions, LR and PSw were more frequently misclassified as Sw, resulting in lower performance for these phases. This misclassification tendency is attributable to the transitional nature of these phases, where LR and PSw are adjacent to Sw and exhibit overlapping acceleration and angular velocity patterns. In contrast, LS and Sw maintained relatively stable classification accuracy despite their larger sample sizes, suggesting that they were less affected by class imbalance. These findings highlight the need to interpret confusion matrices by considering both absolute counts and class distribution, and they reaffirm the common tendency in time-series gait phase classification for misclassifications to concentrate around boundary regions between adjacent phases.

**Table 4:** Test dataset confusion matrix of the CNN.

		**Predicted:**			
		**LR**	**LS**	**PSw**	**Sw**
	**LR**	95.67	0.94	0.14	3.25
	**LS**	5.31	93.16	1.28	0.24
**Actual:**	**PSw**	1.37	1.18	94.41	3.04
	**Sw**	2.08	0.8	5.28	91.84

**Table 5:** Test dataset confusion matrix of the Hybrid LSTM+GRU.

		**Predicted:**			
		**LR**	**LS**	**PSw**	**Sw**
	**LR**	95.28	0.91	0.8	3.02
	**LS**	3.5	94.76	1.56	0.18
**Actual:**	**PSw**	0.16	0.89	97.83	1.12
	**Sw**	2.69	0.57	4.37	92.37

**Table 6:** Test dataset confusion matrix of the transformer.

		**Predicted:**			
		**LR**	**LS**	**PSw**	**Sw**
	**LR**	94.54	1.38	0.79	3.3
	**LS**	3.64	94.93	0.99	0.43
**Actual:**	**PSw**	0.42	2.13	92.87	4.58
	**Sw**	2.16	0.47	2.3	95.07

Table 7 presents the performance comparison of the CNN, Hybrid LSTM+GRU, and Transformer models using hyperparameters optimized with Optuna. Although the Transformer model achieved the highest Macro F1-score of approximately 92.99%, it did not demonstrate a clear performance advantage over the other models, and all three models showed comparable levels of performance. These findings indicate that gait phase classification using an IMUs mounted on the shank can achieve relatively stable and consistent performance across different deep learning architectures.

**Table 7:** Comparison of classification performance across models.

Model	ACC	PR	RC	F1
CNN	93.09	89.11	93.77	91.08
Hybrid LSTM+GRU	94.25	90.61	95.06	92.52
Transformer	94.70	91.86	94.35	92.99

## Discussion

This study investigated gait phase classification using shank-mounted IMUs, and the performance of CNN, Hybrid LSTM+GRU, and Transformer models was compared. The results showed only minor differences among the models, confirming that reliable classification can be achieved even with a single-sensor setup. In contrast, Su et al. [33] attached seven IMUs to the pelvis, thighs, shanks, and feet, and used foot switches to label five gait phases (LR, MS, TS, Psw, Sw). They reported high accuracies with a frame-based deep convolutional neural network, achieving approximately 97% in intra-subject and over 95% in inter-subject evaluations. However, their approach depended on multiple sensors and foot switches, and did not explicitly account for temporal dependencies in the data. By comparison, the present study utilized contact data for labeling without requiring additional devices, and achieved comparable performance with only shank sensors. Although the overall accuracy was slightly lower than that reported by Su et al. [33], the reduced sensor burden and simplified configuration underscore the practical value of our results. These findings indicate that the proposed minimal-sensor approach can reduce system complexity and cost while still maintaining clinically and practically meaningful performance. Furthermore, by evaluating not only CNN but also Hybrid LSTM+GRU and Transformer architectures, this study provides practical guidance on model selection for real-world wearable applications.

A notable finding of this study is that the Transformer model did not outperform the simpler CNN or Hybrid LSTM+GRU models. This can be explained by several factors. First, level-ground gait exhibits highly repetitive and short-range temporal dynamics, and most discriminative information occurs within short local segments. Prior research has shown that healthy gait patterns demonstrate stable and cyclic structure at short temporal scales [46], limiting the potential benefit of long-range dependency modeling. Second, the sliding-window length used in this study was only 20 frames, which restricts the sequence context available to the Transformer. The literature indicates that Transformers show clear advantages primarily when sufficiently long sequences are provided [47]. Third, Transformer architectures typically require large datasets to fully utilize their representational capacity, whereas smaller datasets often favor simpler models. Prior work has demonstrated that complex deep learning models do not necessarily outperform simpler architectures when data are limited [48]. Finally, IMU-based gait signals are dominated by local temporal patterns—such as short-term accelerometer and gyroscope fluctuations—making convolutional and recurrent architectures inherently well suited for this task. Prior studies have noted that Transformer models excel at capturing global dependencies, while CNNs and RNNs can more effectively learn local temporal structures [49]. These factors collectively explain why the Transformer did not exhibit superior performance under the minimal-sensor, short-window setting used in this study.

Gait phases were defined as four categories: LR, LS, Psw, and Sw. Unlike previous studies that separated MS and TS, we integrated the two into a single LS phase, considering both clinical interpretability and research objectives. This simplified four-phase structure was sufficient to capture essential gait-cycle characteristics. In the results, LS and Sw phases were classified with consistently high performance, whereas LR and Psw phases showed greater classification difficulty. Both LR and Psw represent short transitional phases within the gait cycle, where sensor signals are highly variable and boundaries between phases are less distinct. This tendency is consistent with findings in Su et al. [33], where TS exhibited lower detection accuracy, suggesting that transitional phases remain the most challenging regions for classification models.

In terms of training stability, notable differences were observed among the models. The CNN exhibited stable convergence from the early stages of training, suggesting that its relatively simple architecture facilitated easier optimization. In contrast, both the Hybrid LSTM+GRU and Transformer models initially showed considerable fluctuations in loss, which is typical for architectures that directly model temporal dependencies. However, with sufficient training, both models converged stably, and the Transformer demonstrated the ability to capture complex temporal patterns when appropriate training conditions and optimization strategies were applied. These results indicate that CNNs offer strengths in efficiency and practicality, whereas Transformers demonstrate superior capacity in learning more complex patterns—highlighting the importance of selecting models that match the complexity of the application scenario. For simple and highly periodic environments such as level-ground walking, lightweight models like CNNs may be more suitable than excessively complex architectures.

Regarding phase-specific classification tendencies, the LS and Sw phases consistently demonstrated robust performance, whereas LR and PSw were more frequently misclassified as Sw. This pattern likely arises because LR and PSw are short transitional segments in which signal characteristics gradually shift toward those of the adjacent Sw phase. These results reaffirm that transitional phases remain the most challenging segments for time-series–based gait phase classification and suggest the need for more advanced feature extraction strategies capable of enhancing temporally localized transitional characteristics.

This study demonstrated that deep learning–based gait phase classification can be effectively applied even in a simplified phase framework using a minimal sensor configuration, and that clinically and practically meaningful performance can be achieved with only shank-mounted IMUs. The shank provides a stable and consistent attachment site in wearable and robotic applications, making it more reliable than alternatives such as pelvis-mounted sensors, which often suffer from placement variability and motion artifacts. Although deep learning models require higher computational resources during training, this cost is justified by their ability to learn complex temporal patterns and subtle phase-transition dynamics that simpler methods cannot capture, a benefit that has been consistently demonstrated across time-series learning research [50]. In the inference stage, however, techniques such as model quantization can substantially reduce computation time and memory usage, enabling the proposed models to run efficiently on mobile embedded platforms for real-time gait phase detection [51]. Future research should extend the current work beyond level-ground walking in healthy individuals by incorporating diverse environments—such as stairs, irregular terrains, and pathological gait patterns—and by further validating sensor fusion and real-time deployment strategies. Such efforts will enhance the generalization capability and reinforce the utility of the proposed approach as a practical and reliable gait phase classification method under a wide range of real-world conditions.

## Conclusion

In this study, we applied an end-to-end learning approach using time-series data from shank-mounted IMUs to directly predict gait phases, and compared the performance of CNN, Hybrid LSTM+GRU, and Transformer models. The results showed that the overall performance differences among the models were not substantial, and that reliable classification can be achieved even with single-sensor data. In simple environments, lightweight models such as CNNs provided sufficient accuracy, underscoring the importance of selecting model complexity based on the specific requirements of the task. This study was limited to level-ground walking data from healthy individuals. Future work will incorporate more diverse gait environments, such as stairs and irregular terrains, as well as patient populations, to further improve the generalization capability of the proposed models. Through these extensions, the proposed approach is expected to serve as a reliable method for gait phase classification under a broader range of real-world and clinically challenging conditions.
